# Interplay between tumor cells and immune cells of the colorectal cancer tumor microenvironment: Wnt/β-catenin pathway

**DOI:** 10.3389/fimmu.2025.1587950

**Published:** 2025-06-18

**Authors:** Aisha Saleh Janeeh, Khuloud Bajbouj, Bilal Rah, Eman Abu-Gharbieh, Mawieh Hamad

**Affiliations:** ^1^ Department of Clinical Sciences, College of Medicine, University of Sharjah, Sharjah, United Arab Emirates; ^2^ Research Institute for Medical and Health Sciences, University of Sharjah, Sharjah, United Arab Emirates; ^3^ Department of Basic Medical Sciences, College of Medicine, University of Sharjah, Sharjah, United Arab Emirates; ^4^ Department of Biomedical Sciences, School of Veterinary Medicine, University of Pennsylvania, Philadelphia, PA, United States; ^5^ Biopharmaceutics and Clinical Pharmacy, Faculty of Pharmacy, The University of Jordan, Amman, Jordan; ^6^ Department of Medical Laboratory Sciences, College of Health Sciences, University of Sharjah, Sharjah, United Arab Emirates

**Keywords:** Colorectal cancer, Wnt/β-catenin pathway, tumor microenvironment, macrophages, immune infiltration

## Abstract

Colorectal cancer (CRC) is currently ranked as the third most frequent human cancer and the fourth leading cause of cancer-related deaths worldwide. Macrophages and immune cell subsets infiltrate the tumor microenvironment (TME) and modulate several cellular events and metabolic processes in CRC. Therefore, CRC-TME-infilitrating macrophages are thought to play a significant role in CRC progression, and could hence be potential therapeutic targets in CRC. Several lines of evidence suggest that the Wingless/Integrated (WNTs) family of signaling proteins plays a crucial role in CRC development and progression. Numerous studies have established that Wnt pathway signaling is involved in CRC-TME interaction; CRC-immune cell interaction in particular. Mounting experimental evidence point to the possibility that the TME in CRC can reciprocally modulate the Wnt/β-catenin pathway. Lastly, several studies have elaborated on the effect of drugs that disrupt the Wnt/β-catenin pathway as means of hindering CRC growth and progression. In this review, we discuss the multifaceted role of Wnt/β-catenin pathway in CRC and its TME as well as CRC-TME interactions. We also elaborate on the potential therapeutic utility of Wnt/β-catenin pathway-related targets in CRC.

## 1 Introduction

CRC is ranked as the third most common cancer worldwide, with a death rate of about 5.8%. Globally, about 600,000 new cases were reported by the end of 2020 (1). Typically, CRC requires a significant amount of time to manifest as diagnosable cancer; usually 10 to 20 years. Various factors contribute to disease onset and progression with the accumulation of inherited epigenetic and genetic alterations being of the utmost importance. CRC can arise through distinct pathways, making it particularly intriguing. The intricate interplay between genetic mutations, epigenetic changes and other factors contribute to disease complexity and diversity (2). Numerous modifiable and non-modifiable risk factors contribute to CRC development (3). Current treatment approaches of CRC include chemotherapy, targeted therapy, and immunotherapy (4).

Wnt genes, which were first described in 1980 as analogues of the wingless gene in fruit flies, have since collectively emerged as an important player in a wide range of cellular processes including cell polarization, proliferation, differentiation, and migration during embryonic development and tissue repair processes (5). The Wnt signaling pathway comprises the canonical pathway and the non-canonical pathway. This branching allows for intricate and diverse cellular responses to Wnt ligands depending on the specific context and signaling cues involved (6). Mounting evidence suggest that the Wnt/β-catenin signaling pathway plays a dual role in the TME and CRC genesis and progression. A diverse set of immune cells infiltrate the CRC-TME, which envelopes malignant cells, mainly macrophages (7). Tumor-associated macrophages (TAMs) polarize into either inflammatory anti-tumorigenic (M1) or anti-inflammatory pro-tumorigenic (M2) cells (8).

This review uniquely integrates the roles of the Wnt/β-catenin pathway and immune cells within the CRC microenvironment, offering a comprehensive analysis of how this signaling modulates immune cell functions and interactions. It highlights novel mechanistic insights into the dual roles of Wnt/β-catenin in promoting immune evasion and tumor growth while also discussing potential therapeutic strategies targeting this pathway to enhance anti-tumor immune responses.

## 2 Basic biology and clinical aspects of CRC

### 2.1 CRC carcinogenesis

The alarming rise in CRC cases and mortality over the last few decades emphasizes the need for further research of the basic biology of CRC as means of improving disease diagnosis and treatment (9). Only 5% of CRC cases are hereditary, and it usually develops spontaneously due to adenomatous polyposis coli (APC) mutations. APC is a tumor suppressor gene that participates in the Wnt/β-catenin signaling pathway by regulating β-catenin concentration (10). Activation of the Kristen Rat Sarcoma viral oncogene (KRAS) occurs in around 30-40% of CRC cases, this allows for about 10% of adenomatous polyps to develop into adenocarcinomas (11). Subsequent loss of the tumor suppressor gene “deleted in colorectal cancer” (DCC) causes the tumor to enlarge and penetrate the serosa (stage II) (12). Loss of tumor suppressor gene p53 that arises in about 50% of CRC cases allows the tumor to grow and proceed to penetrate the visceral peritoneum (stage III) (13). Additional mutations, including mutations in the Wnt/β-catenin signaling pathway, increase the risk of lymphatic or blood vascular metastases (advanced or stage IV CRC). Cancer cells metastasize to different body organs, including lungs, bone and liver (14).

### 2.2 CRC risk factors

Onset and progression of CRC have been lined to several risk factors including both modifiable and non-modifiable ones. Among the modifiable risk factors is obesity which promotes insulin resistance, hyperinsulinemia, and high levels of insulin-like growth factor-1 (IGF-1); all of which stimulate cell proliferation (15). Alcohol metabolism entails converting ethanol to its metabolites, which have the potential to exert significant carcinogenic effects in the colon. The colon microbiota may also contribute to CRC progression through multiple means including the synthesis of alcoholic metabolites (16). Non-modifiable risk factors in hereditary CRC include germline mutations that lead to Lynch syndrome and familial adenomatous polyposis (FAP). DNA mismatch repair (MMR) gene mutations in the MSH2, MLH1, PMS2, MSH6, or epithelial cell adhesion molecule (EpCAM) are the main genetic causes of Lynch syndrome FAP is a rare genetic disorder that is precipitated by genetic mutations in the APC gene (17).

### 2.3 CRC treatment approaches

Chemotherapy, targeted therapy, and immunotherapy are among the most promising approaches in CRC patient management (18). 5-Flurouracil (5-FU) was one of the first and most widely used drugs in cancer patients. It primarily works by inhibiting thymidylate synthase (TS), which disrupts the intracellular deoxynucleotide pool that sustains DNA replication (19). The anti-tumor activity of 5-FU ensues after it interacts with phosphorylated sugars through enzyme-catalyzed reactions that form active metabolites including FdUMP, FdUTP and FUTP (20). Irinotecan, a semi-synthetic form of the natural alkaloid camptothecin, is another drug with a therapeutic utility in CRC. The drug functions as a DNA topoisomerase I inhibitor and causes single-strand DNA (ssDNA) breaks. Topoisomerase I inhibitors limit DNA re-ligation, which results in cell death, by stabilizing intermediate cleavage complexes generated between the enzyme, the inhibitor, and DNA strands (21). The third-generation platinum drug oxaliplatin, which received full approval from the US FDA in 2004 for the treatment of advanced or metastatic CRC when combined with 5-FU, causes cross-linking between and within DNA strands; it also induces ssDNA and dsDNA breaks, thus preventing replication and transcription (22). Cetuximab, a humanized murine monoclonal IgG, is used as a targeted therapy to specifically target the extracellular domain of the epidermal growth factor receptor (EGFR). It attaches to the EGFR’s extracellular domain and blocks ligand binding, thus inhibiting ligand phosphorylation and internalization and hence hindering cell survival and growth (23). The FDA granted cetuximab approval to treat metastatic CRC, either as monotherapy or in combination with irinotecan in 2004 (24). Bevacizumab, a recombinant humanized monoclonal antibody, is used as a targeted therapy for CRC as it specifically targets the isoforms of vascular endothelial growth factor A (VEGF-A), a ligand that binds to VEGF receptors 1 and 2 (25). Checkpoint inhibitors such as ipilimumab (CTLA4-CD80/86 inhibitor) and nivolumab (PD1-PDL1/2 inhibitor) have also garnered significant attention in recent years. CTLA4 is a co-inhibitory molecule that, once engaged with CD80 and CD86 on antigen presenting cells, inhibits T-cell activation. Therefore, inhibiting CTLA4 engagement by blocking antibodies allows for T cell activation and tumor cell recognition and destruction. T cell-mediated tumor cell killing was also shown to be achievable by the use of monoclonal antibodies that block the interaction between PD-1 on T cells and PD-L1 on tumor cells (26). The availability of several potent drugs to deal with CRC notwithstanding, chemoresistance remains a significant concern. In that, about 90% of metastatic CRC cases are thought to experience treatment failure due to chemoresistance, whether inherent or acquired (27). Resistance to conventional cytotoxic therapy can manifest as reduced drug delivery, especially in the case of large volume tumors where blood supply, oxygenation and nutritional supplement deliver are also restricted. Drug dose escalation may temporarily overcome this problem but, long term, it tends to precipitate significant hepato- and or nephrotoxicity (28). Resistance to targeted therapy is caused by a variety of genetic processes, including mutations that result in the loss of some tumor suppressor genes, such as P53, as well as the overexpression of some oncogenes, such as BCR-ABL and HER2, or the stimulation of downstream signaling molecules within specific pathways (29).

## 3 Wnt/β-catenin pathway

Engagement of the Wnt protein with one of the Wnt ligands, a family of 19 endoplasmic reticulum (ER)-derived glycoproteins, generate an array of signals that operate in a paracrine or an autocrine manner. Wnt signaling pathway operates in a β-catenin-dependent canonical manner or in a β-catenin-independent noncanonical manner (30).

### 3.1 The canonical Wnt/β-catenin signaling pathway

**Figure 1:**
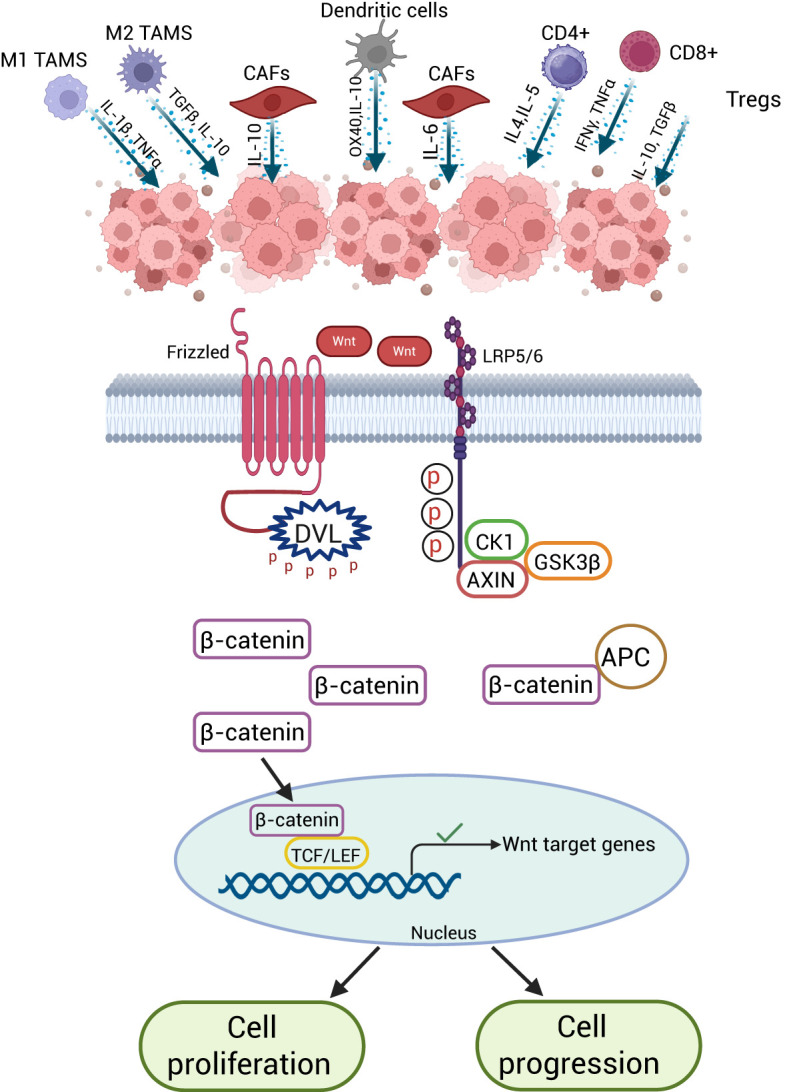
Basics of the canonical Wnt signaling pathway. **(A)** In the absence of Wnt ligand, β-catenin is tagged for phosphorylation by the destruction complex (APC, AXIN, CK1, GSK3β) and proteasomal degradation. **(B)** In the presence of Wnt ligand, β-catenin escapes degradation, accumulates in the cytoplasm and translocates to nucleus to activate Wnt target genes. Created with BioRender.com.

The canonical Wnt/β-catenin pathway is primarily involved in regulating cell proliferation and differentiation. Disrupted canonical Wnt/β-catenin signaling may associate with abnormal cell proliferation and/or differentiation and tumorigenesis. In the presence of Wnt ligands, the canonical pathway is initiated through the lipoprotein receptor-related protein (LRP5/LRP6)/Frizzled (FZD) receptor (**Figure 1**). FZD receptor interacts with the cytoplasmic protein dishevelled (DVL), which operates upstream of β-catenin and glycogen synthase kinase 3 β (GSK3β). This stabilizes the β-catenin and prevents its phosphorylation, ubiquitination and proteasomal degradation. This allows it to accumulate and translocate to nucleus, where it attaches to the N-termini of DNA-binding proteins of the T-cell factor (TCF)/lymphoid enhancer binding factor (LEF) family to regulate target gene expression (31). In the absence of wnt signaling ligands however, the transcriptional co-activator β-catenin is inactive due to GSK-3-mediated phosphorylation. A multimeric protein complex named the destruction complex, which consists of APC, casein kinase 1 (CK1), GSK3β, axis inhibitor (Axin) is responsible for the ubiquitination of β-catenin by an E3 ubiquitin ligase (β-TrCP) and its subsequent proteasomal degradation. This allows for the repressor complex TCF/LEF and transducing-like enhancer protein (TLE)/Grouche to engage and represses target gene transcription. Subsequent to the binding of Wnt to FZD-Axin-LRP-5/6 complex, cytosolic GSK-3β is sequestered and hence β-catenin phosphorylation and degradation is blocked (32).

### 3.2 The noncanonical Wnt pathway

**Figure 2:**
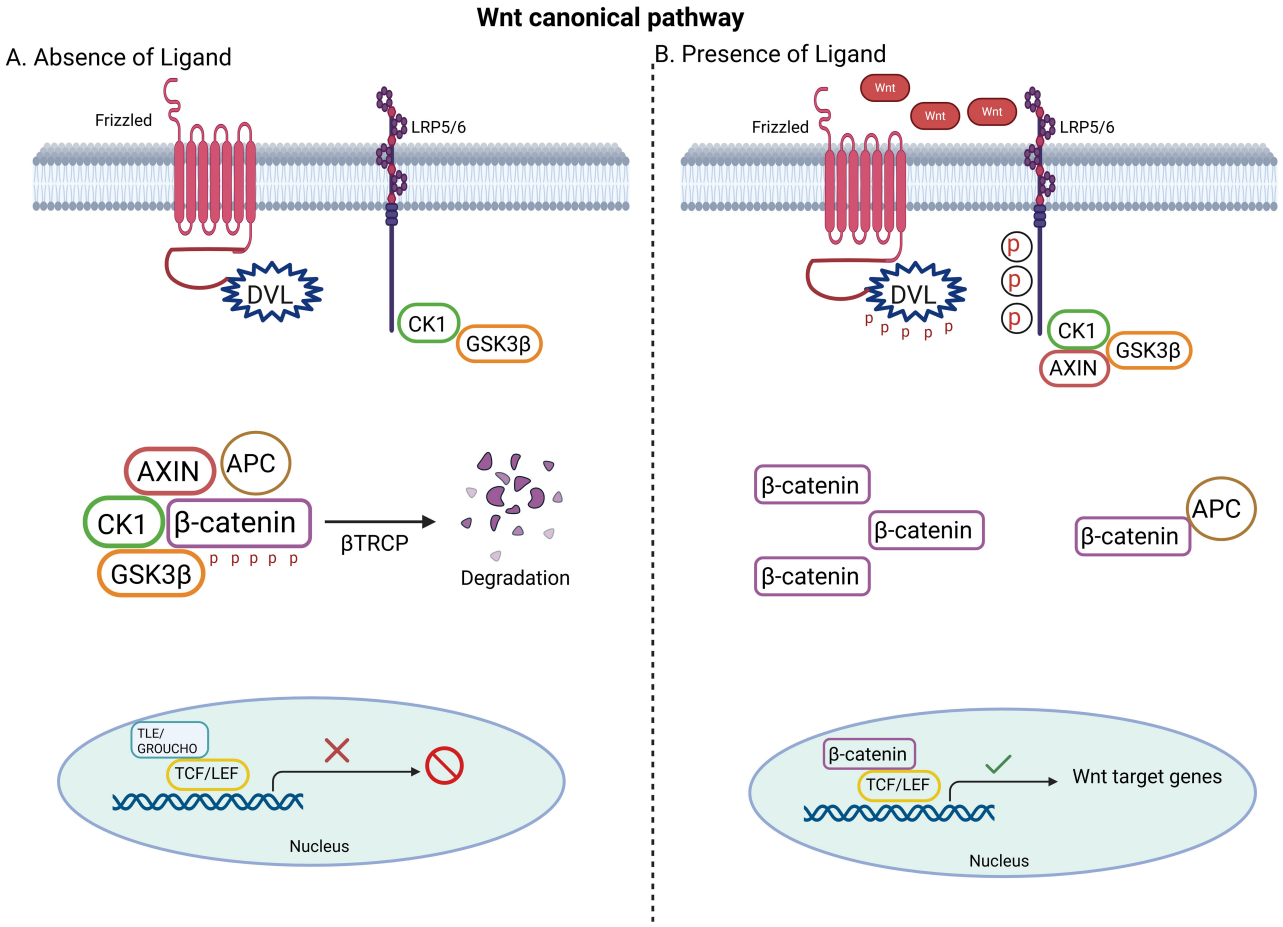
Basics of the noncanonical Wnt signaling pathway. **(A)** In the presence of Wnt ligands, Wnt/PCP signaling involves the binding of Wnt to ROR-FZD receptor complex and results in the activation of DVL. The activated DVL generates the activation of Rho by the de-inhibition of cytoplasmic protein DAAM. Rac1 and Rho together provoke ROCK and JNK and advocate polarized cell migration **(B)** In the presence of Wnt ligands, Wnt/Ca^2+^ pathway involves the activation of PLC to produce DAG and IP3, which lead to intracellular Ca^2+^ flux that recruits PKC, calcineurin and CAMKII to form transcription factors such as NFκB and NFAT. Created with BioRender.com.

This β-catenin-independent Wnt pathway involves several receptors that mediate cellular signaling. For example, Wnt/planar cell polarity (PCP) signaling pathway consists of FZD and Wnt transmembrane receptor proteins (**Figure 2**). Wnt11, Wnt5B, and Wnt5A translate PCP signals through the receptors FZD6 or FZD3 and co-receptors Tyrosine-protein kinase transmembrane receptors (ROR1 and ROR2). The FZD receptor initiates a signaling cascade that involves the small Rho family GTPases Cdc42, Rac, and Rho, along with Jun-N-terminal kinase (JNK) (33). The PCP pathway promotes tissue patterning and morphogenesis; a crucial feature of epithelia that are defined by apical-basal polarization within the epithelium plane. It can also control polarity in non-epithelial cells at the cell migration level (34). The FZD protein is coupled to trimeric G protein, which engages phospholipase C (PLC) and hence the Wnt/Ca^2+^ pathway. This pathway involves several Ca^2+^-related proteins including calcium calmodulin-dependent protein kinase II (CaMKII), inositol 1 4,5-triphosphate (IP3), and 1,2 diacylglycerols (DAG). The Wnt/Ca^2+^ pathway recruits and activates several transcription factors including the nuclear factor associated with T-cells (NFAT), cAMP-response element binding protein (CREB), and NFκB to target and upregulate the expression of related gene sets. The interaction between Wnt/FZD receptor 2 ligand might stimulate the phosphodiesterase 6 (PDE6) in a Ca^2+^-dependent manner, leading to decreased cGMP production. The Wnt/Ca^2+^ pathway has been linked to neurological disorders, inflammation, and malignancy (35).

### 3.3 Wnt pathway signaling in CRC

In sporadic CRC, mutated APC gene was documented in >80% of cases of FAP syndrome (36). Other cases may be attributed to mutations in β-catenin and/or Axin genes (37). The growth of CRC is significantly influenced by enhanced transcription of cyclin D1 through RAS and β-catenin (38). About 90% of CRC patients express MMP-7, which is another target for β-catenin/TCF signaling; this target is linked to increased morbidity and mortality in CRC (39). Unsurprisingly, the association between CRC and abnormal Wnt/β-catenin signaling demonstrates that Wnt/β-catenin inhibitors can be potential targets for treatment of CRC. Besides, abnormalities in oncogenes that regulate the activity of β-catenin can indirectly contribute to CRC. About 60% of CRC cases have CDK8 (cyclin-dependent kinase-8) gene amplification, another genetic alteration that modify β-catenin activity; higher CDK8 levels stimulate neurogenic locus notch homolog protein 1 (Notch1) and β-catenin to promote cell differentiation (40).

### 3.4 Wnt pathway components as potential therapeutic targets in cancer

Currently, few compounds that specifically target the Wnt pathway are available; none has been FDA-approved as anti-cancer agents. Although the role of Wnt signaling in cancer development, progression and metastasis is broad and significant, Wnt signaling, being highly complex and diverse, could be a significant obstacle in developing effective anti-cancer agents that effectively disrupt it. The discussion below outlines some novel anti-cancer therapies that target specific Wnt pathway members.

Porcupine (PORCN), a membrane-bound O-acyltransferase, aids in the post-translational palmitoylation of Wnts members to facilitate their trafficking, secretion, and activity (41). LGK974 (WNT974), a PORCN inhibitor, was reported to promote the remission of Wnt-related cancers in rats and was thus moved along for clinical testing in patients (42).

ETC-159, an oral selective small molecule inhibitor of porcupine that inhibits Wnt protein (all members) secretion, is another PORCN inhibitor that has demonstrated significant anti-cancer effect in preclinical investigations and has thus been entered into phase 1 clinical trials (43). Treatment of RSPO-translocated CRC patient-derived xenografts with ETC-159 in mice was reported to precipitate significant anti-growth effects (44).

## 4 Immune cells within the CRC-TME

**Figure 3:**
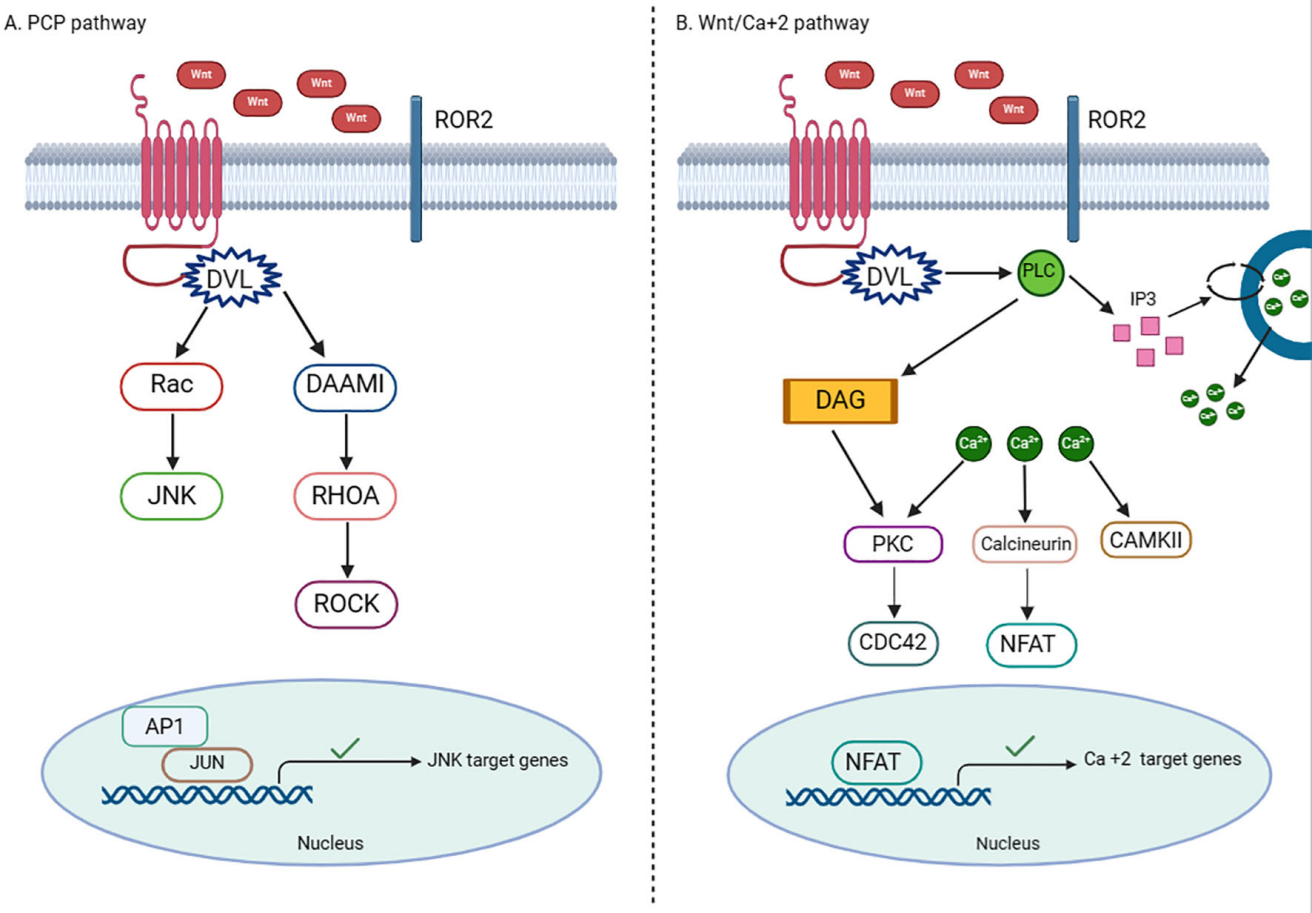
The CRC immunosuppressive microenvironment. The colorectal cancer (CRC) tumor microenvironment (TME) is comprised of cancer cells, immune cells, blood vessels, and fibroblasts. Cancer cells have the ability to modulate the TME to enhance immune evasion, aggressiveness, invasiveness, and metastatic potential. Key immune cells within the TME include CD8⁺ T-cells, CD4⁺ T-cells, regulatory T-cells (Tregs), dendritic cells, and tumor-associated macrophages (TAMs), which can exist in either a pro-inflammatory (M1) or immunosuppressive (M2) state.

Numerous studies have established that the TME is essential for the growth and metastasis of cancer. Beside the offending tumor cells, the TME consists of several cell types including fibroblasts, immune cells, and endothelial cells, which collectively secrete and express numerous metabolites and ligands that interfere with tumor mass metabolism, growth and immune evasion (**Figure 3**).

### 4.1 T helper cells

Several CD4^+^ T helper (Th) cell subsets with varying effects on tumor growth have been identified in the CRC-TME. For example, Th1 cells tend to enhance T cytotoxic (Tc) effector function and are hence thought to mediate anti-tumor immune responses. Overexpression of Th1 cluster genes has been associated with greater better disease-free survival in CRC patients (45). In contrast, increased numbers of Th17 within the CRC-TME were reported to associate with suppressed immunity and poor patient survival. This is in line with other preclinical studies which have shown that Th17-derived cytokines including IL-17, IL-21, and IL-22 promote, rather than hinder, CRC cell growth (46).

### 4.2 Dendritic cells

Dendritic cells (DCs) are professional antigen presenting cells that present antigens to Th cells. Cancer cells often suppress DCs by, for example, releasing immunosuppressive cytokines such as the transforming growth factor-beta (TGF-β). In CRC, myeloid-derived DCs, the most prevalent DC subtype in lymph nodes invading CRC were shown to induce Th1 polarization (47). Compared with DCs from healthy counterparts, DCs derived from CRC patients exhibit poor antigen presentation potential and decreased T cell co-stimulatory activity. DCs from CRC patients tend also to release greater quantities of the immunosuppressive cytokine IL-10 and smaller amounts of the immunostimulatory cytokines IL-12 and tumor necrosis factor-alpha (TNF-α) (48). Recent studies have shown that CXCL1 is highly expressed in CRC patient-derived DCs and that such cells are able to promote tumor cell motility, epithelial mesenchymal transition (EMT) and cancer cell stemness (49). This suggests that the CRC-TME may reduce DC’s immunostimulatory potential and induce their immunosuppressive behavior.

### 4.3 Regulatory T cells

Tregs, which conventionally co-express forkhead boxP3 (FoxP3) and CD25, tend to associate with immunosuppression, metastases, and poor disease prognosis. As a general case, Tregs regulate immune responsiveness as means of fending off against autoimmunity through their ability to secrete adenosine, block IL-2 production, and release immunosuppressive cytokines such as IL-35, TGF-β, and IL-10 (50). The role of Tregs in the CRC-TME is complex and ambiguous; several studies have shown that Tregs exert both pro- or anti-tumor effects, depending on the precise phenotypic profile of the Tregs involved. In that, high rates of Treg infiltration were shown to positively correlate with poor prognosis based on a study which involved 81 pretreatment biopsies from patients with locally-advanced CRC (51). In contrast, some studies have shown that increased FoxP3^+^ cell infiltration associates with increased survival. As noted earlier, Treg heterogeneity may be at work here; it was previously reported that naive Tregs (FoxP3^low^/CD45RA^+^) in CRC specimens are either pro-inflammatory (FoxP3^low^/CD45RA^-^) or immunosuppressive (FoxP3^high^/CD45RA^-^) with terminal differentiation (52).

### 4.4 Cytotoxic T cells

Tc cells are the main cellular effectors of the adaptive immune response and are thought to play a pivotal anti-tumor role. The presence of Tcs in patient-derived CRC tumor cores was shown to associate with reduced risk of recurrence (53). Therefore, tumor cells have evolved several mechanisms to block or reduce Tc activity as means of evading the adaptive immune response. For instance, TNF-α-mediated inflammation in CT26 CRC cells induced the synthesis of PD-L1 on stromal cells, which engaged the inhibitory T cell marker PD1 and the inhibition of CD8^+^ T cell activation (54).

### 4.5 Cancer-associated fibroblasts

Several studies have suggested that increased infiltration of cancer-associated fibroblasts (CAFs) associate with ECM remodeling, angiogenesis, invasion, desmoplasia, immunosuppression, and chemoresistance (55). Using single-cell RNA sequencing approaches, two subpopulations of CAFs were identified in the CRC-TME; CAF-A, which expresses ECM remodeling-associated genes such as COL1A2, MMP2 and DCN and CAF-B, which are myofibroblast-like cells (56). Previous work has demonstrated the presence of an adverse relationship between Th1/Th0 ratio and the average expression of CAF-specific genes in microsatellite stable CRC samples (57). Release of CXCL8 by CAFs, which recruits monocytes to the CRC-TME and promotes macrophage M2 phenotype polarization could also contribute to the immunosuppressive state of the TME. In this respect, CAFs were reported to promote metastasis in a variety of malignancies including CRC (58). Greater than 78% of CRC clinical samples exhibit desmoplasia that is attributed to the ability of CAF to produce ECM remodeling enzymes, collagen, and other cytoskeletal proteins (59). CRC cell-dependent activation of TGF-β/Smad2 signaling in CAFs results in upregulated expression of α-SMA, which is consistent with a differentiated Myo fibroblastic phenotype that promotes the production of several MMPs and other invasion-related proteinases (60). In short, CAFs seem to support CRC growth and progression by suppressing key immune cell subsets, remodeling the extracellular matrix, and inducing EMT.

### 4.6 Tumor-associated macrophages

Macrophages encompass a variety of immune cell subsets with a diverse set of functions in host defense against infections, tissue architecture and tissue repair and homeostasis (61). Macrophages that infiltrate the TME, also known as TAMs, polarize into an M1 anti-tumorigenic phenotype or a tumor-promoting M2 phenotype. While activated M1 cells produce proinflammatory cytokines such as IL-1, IL-6, and TNFα, stimulated M2 TAMs may contribute to tumor angiogenesis along with other TME cells by releasing VEGF. M2 TAMs also the ability of tumor cells to evade adaptive immunity by secreting the immunosuppressive cytokines IL-10 and TGF-β. Furthermore, TAMs secrete MMP-9, an essential proteolytic enzyme that assists in the rearrangement of the ECM in several cancers including CRC. The assertion that the M1 phenotype is generally recognized as being an anti-tumorigenic phenotype, is contradicted by the observation that M1-like macrophages were reported to promote and sustain chronic inflammation in animal models of induced colitis, which itself increases the risk of CRC (6). Supplementation of conditioned media with M2-derived metabolites and secretions was shown to enhance cell proliferation and induce the expression of anti-apoptotic markers (survivin and BMI-1) in CRC cell lines. In contrast, supplementation of cultured CRC cells with M1-derived metabolites and secretions reduced CRC cell viability and enhanced apoptosis (62). Activated M2 TAMs also produce considerable amounts of the angiogenic factor *VEGF*. Transcriptome- and proteome-based work on CRC M2 TAMs has shown that these cells are enriched for molecules related to ECM reorganization (63). TAMs were previously shown to activate the JAK2/STAT3 pathway through IL-6, inhibit tumor suppressor miRNAs like R-506-3p and its target FoxQ1, secrete CCL22, and activate the PI3K/AKT pathway (64). These TAM related effects coupled with the ability of CRC cells to produce IL-10 through STAT3 signaling promotes tumor growth, invasiveness, and aggressiveness (65).

### 4.7 Extracellular matrix of the CRC-TME

Proteoglycans, collagen, glycosaminoglycans and hyaluronic acid are among the major constituents of the extracellular matrix (ECM) that are subject to structural and/or functional alterations within the TME. ECM serves as a physical framework for tumor cells and actively supports paracrine signaling, cell-cell adhesion, metastasis, immune evasion and tumor growth (66). Recent work has shown that the concentration of the remodeling enzyme MMP-9 was significantly higher in the CRC-TME as compared with healthy mucosa and that this associated with poor prognosis. Increased production of the ECM-degrading enzyme MMP-2 has also been reported to associate with advanced CRC and lymphoid tissue invasion (67).

## 5 Wnt/β-catenin at the interface of CRC-TME immune cell interactions

Numerous previous studies have elaborated on the role of Wnt/β-catenin in immune response development and function. It is well accepted now that Wnt/β-catenin signaling plays an important role in regulating the expression of several immune response-related genes and is therefore believed to modulate the activation of several immune cell subsets (68). In that, it was reported to regulate the development and differentiation of various lymphocyte subsets including Tregs (69). Wnt/β-catenin signaling was also linked to the maintenance of immune tolerance, mainly through Treg regulation (70). It was also reported to modulate DC inflammatory responses by inducing the production of pro-inflammatory cytokines and chemokines. In fact, dysregulated Wnt/β-catenin signaling has been implicated in the development and progression of immune system cancers (71).

Regarding the question of whether the Wnt/β-catenin is involved in the immune responsiveness within the CRC-TME, previous work has shown that Wnt/β-catenin pathway activation promotes immunosuppression, immune evasion, immune cell infiltration, and immune checkpoint regulation among other processes that favor CRC growth and metastasis. For example, previous work has demonstrated that activation of the Wnt/β-catenin pathway in CRC tumors associates with increased immuno-evasive behavior mainly through its ability to promote the suppression of antitumor immune responses and hence allowing CRC cells to evade immune surveillance and clearance (72). T cells and NKs were shown to be key targets for the immunosuppressive effects of the Wnt/β-catenin pathway in CRC-TME (73). Aberrant Wnt/β-catenin signaling in CRC tumors was also reported to negatively affect the recruitment and distribution of various immune cell subsets into the CRC-TME (74). Wnt/β-catenin pathway activation was also implicated in disrupting the protective expression pattern of immune checkpoint proteins including PD-L1 (75). Therefore, targeting Wnt/β-catenin pathway components could potentially enhance the antitumor immune response and sensitize CRC cells to immunotherapy.

## 6 Conclusion

**Figure 4:**
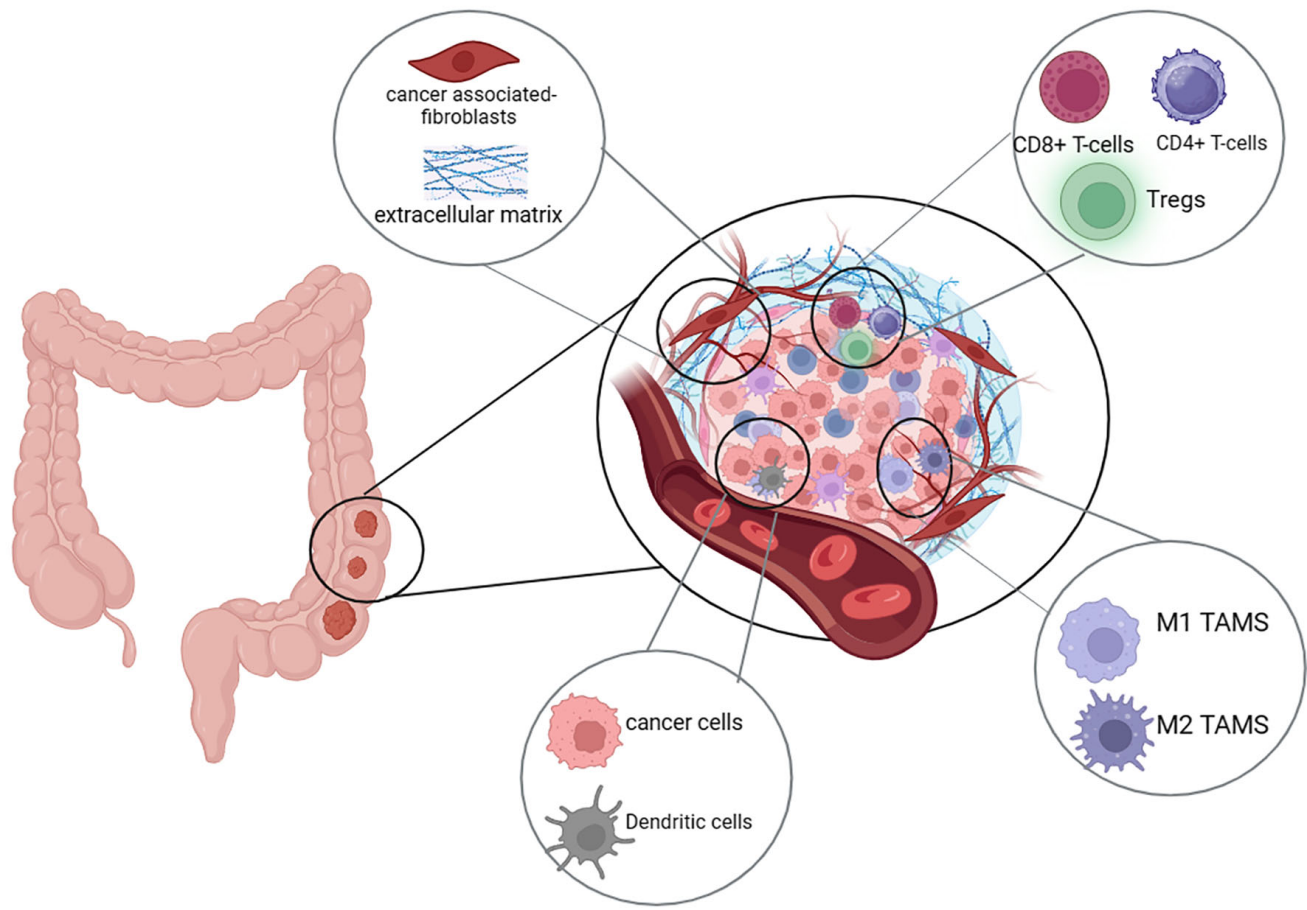
Wnt/β-Catenin signaling in the CRC-TME. Aberrant Wnt/β-catenin signaling in colorectal cancer cells promotes tumor growth, ECM remodeling by CAFs, and immune evasion. It reduces CD8⁺ and CD4⁺ T cells, suppresses M1 macrophages and dendritic cells, and enhances Tregs and M2 macrophages, creating an immunosuppressive microenvironment.

This review provides valuable insights into CRC, covering its origin, mutation, and the challenges of therapy resistance. It also focuses on the role of Wnt/β-catenin in CRC along with recent therapeutic advances. Additionally, it highlights the significant role of Wnt/-β-catenin in shaping the CRC-TME, which plays crucial roles in tumor development, immune evasion, metastasis, and invasion. **Figure 4** summarizes the interaction between Wnt/β-catenin signaling and the CRC tumor microenvironment, demonstrating how these interactions promote tumor cell proliferation and progression within the TME. Wnt signaling also appears to be critical in promoting tumor cell proliferation and progression within the TME. Wnt signaling is required for the formation and proliferation of hematopoietic and immune cells, but its regulation within the tumor microenvironment appears to create a context-dependent environment that impairs immune cells’ ability to mount an effective response by preventing tumor antigen presentation. Lastly, it is clear that signaling through both the canonical and the non-canonical Wnt pathways seems too operative in CRC. This suggests that a variety of Wnt/β-catenin-related molecules can be targeted for possible therapeutic intervention in CRC.
